# Recurrent combined branch retinal vein and branch retinal artery occlusion associated with PAMM as the first manifestation of lung adenocarcinoma: a case report

**DOI:** 10.1186/s12348-026-00574-1

**Published:** 2026-03-31

**Authors:** Eya Essassi, Wijden Nabi, Aymen Abdeljelil, Melek Kechida, Sana Khochtali, Bechir Jelliti, Moncef Khairallah

**Affiliations:** 1 Department of Ophthalmology, Faculty of Medicine, Fattouma Bourguiba University Hospital, University of Monastir, Monastir, 5019 Tunisia; 2 Department of Ophthalmology, Faculty of Medecine, Aghlabites Hospital of Kairouan, University of Sousse, Sousse, Tunisia; 3 Department of Internal Medicine and Endocrinology, Faculty of Medicine, Fattouma Bourguiba University Hospital, University of Monastir, Monastir, Tunisia

**Keywords:** Antiphospholipid syndrome, Branch retinal artery occlusion, Branch retinal vein occlusion, Lung adenocarcinoma, Paracentral acute middle maculopathy

## Abstract

**Purpose:**

To report a case of recurrent bilateral combined branch retinal vein occlusion (BRVO) and branch retinal artery occlusion (BRAO) with paracentral acute middle maculopathy (PAMM) as the first manifestation of lung adenocarcinoma.

**Case report:**

A 54-year-old male with a history of inferonasal BRVO in the left eye (LE), was referred to our department for blurred vision in the LE. On examination, best-corrected visual acuity was 20/20 in the right eye (RE) and 20/25 in the LE. Fundus examination showed tortuosity and dilatation of the inferotemporal branch vein, cotton-wool spots, retinal hemorrhages, and areas of retinal whitening in both eyes corresponding to a BRVO associated with BRAO and PAMM. A few months later, the patient complained of blurred vision in the RE. Examination revealed a superior temporal BRVO and BRAO. An exhaustive work-up was performed. Results showed a high level of antiphospholipid antibodies. Chest scan showed the presence of a pulmonary mass related to lung adenocarcinoma. The patient underwent a surgical removal of the lung tumoral mass and chemotherapy. The level of antiphospholipid antibodies was back to normal and no recurrence of ocular symptoms was noted after a follow-up of one year.

**Conclusion:**

The association between retinal vascular occlusions and systemic carcinomas is rare. Malignant tumors may induce a hypercoagulation state and an increased risk of thromboembolic complications including branch retinal vascular occlusions.

## Introduction

Retinal vascular occlusions, broadly categorized into retinal vein occlusions and retinal artery occlusions are a leading cause of visual loss after diabetic retinopathy. They are most commonly associated with advancing age, hypertension, cardiovascular diseases, and other systemic factors such as diabetes, hyperlipidemia, and smoking [1]. Rare causes of retinal vascular occlusions include hypercoagulable states, hyperviscosity syndromes, and infectious and noninfectious vasculitic diseases [2]. Retinal vascular occlusions have been rarely associated with a known or occult systemic malignancy [3].

We herein describe a 54-year-old male with recurrent bilateral combined branch retinal vein occlusion (BRVO) and branch retinal artery occlusion (BRAO) who ultimately was diagnosed with lung adenocarcinoma associated with antiphospholipid syndrome (APS).

## Case report

**Fig. 1:**
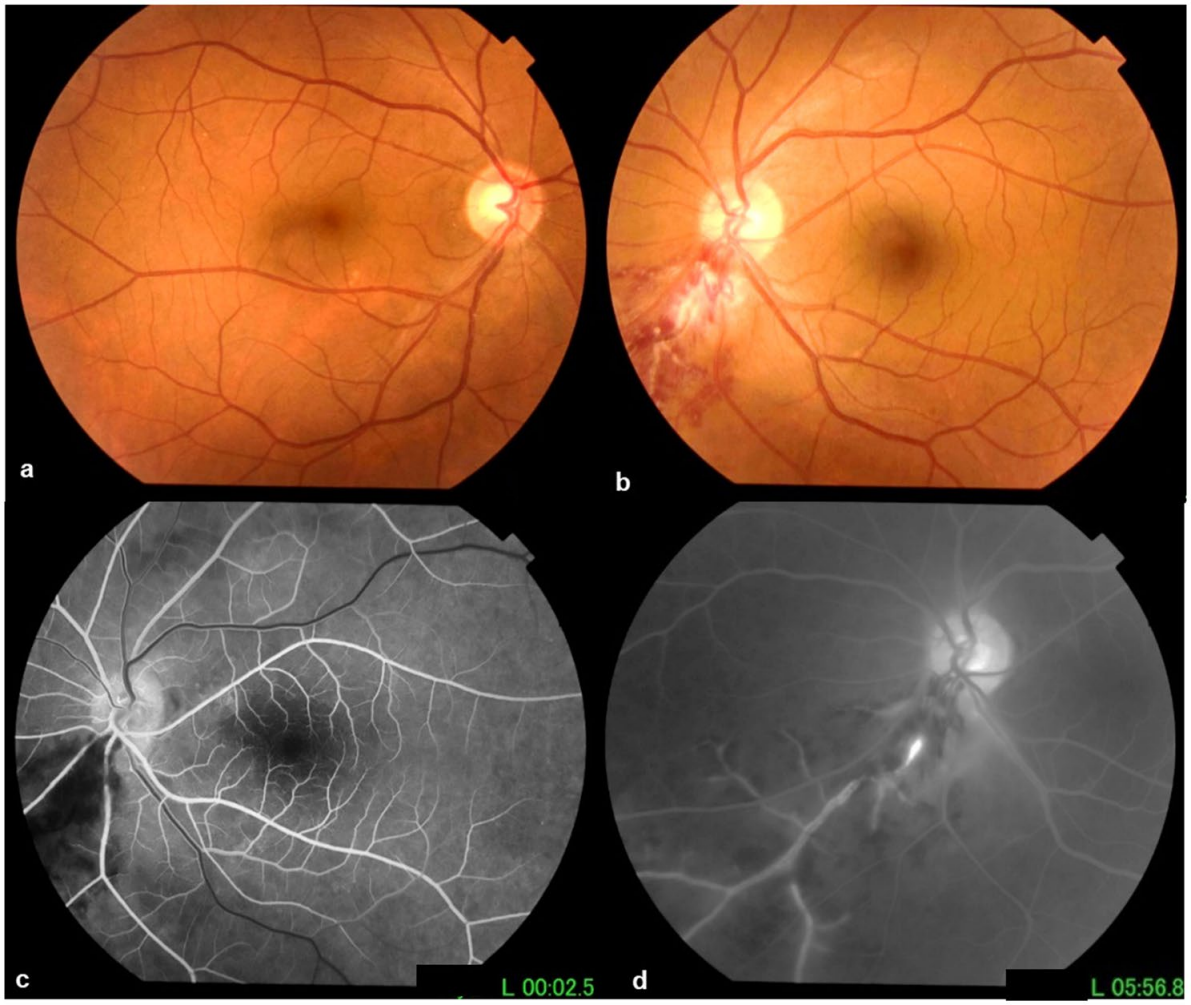
**(a)** Fundus photograph of the right eye showing no abnormalities. **(b)** Fundus photograph of the left eye showing superficial and deep retinal hemorrhages, cotton-wool spots, and retinal vascular sheathing inferonasally. **(c)** Early-phase fluorescein angiogram of the left eye showing delayed filling of the inferonasal retinal vein branch and hypofluorescent areas by masking effect from retinal hemorrhages. **(d)** Late-phase fluorescein angiogram of the same eye showing retinal vein dilatation and tortuosity and retinal vein staining and leakage

A 54-year-old male presented to our department with sudden and painless vision loss in the LE. He was then a current smoker, having accumulated 42 pack-years and reported a history of inferonasal BRVO documented with fluorescein angiography (FA) in the left eye (LE) three months earlier (Fig. 1). At presentation to our department, best-corrected visual acuity (BCVA) was 20/20 in the right eye (RE) and 20/32 in the LE. No abnormalities were noted in the anterior segment on slit-lamp examination in both eyes. Intraocular pressure was 12 mmHg. Fundus examination showed dilated and tortuous inferotemporal branch retinal vein with associated cotton-wool spots and areas of ischemic retinal whitening in the posterior pole in both eyes and flame-shaped and blot retinal hemorrhages in the LE. FA showed a marked delay in retinal arteriovenous transit time and venous filling, with a sluggish flow appearance and optic disc hyperfluorescence in the late phase, in the affected areas of both eyes. OCT scan through the whitish retinal lesions showed thickening and hyperreflectivity of the inner retinal layers.

**Fig. 2:**
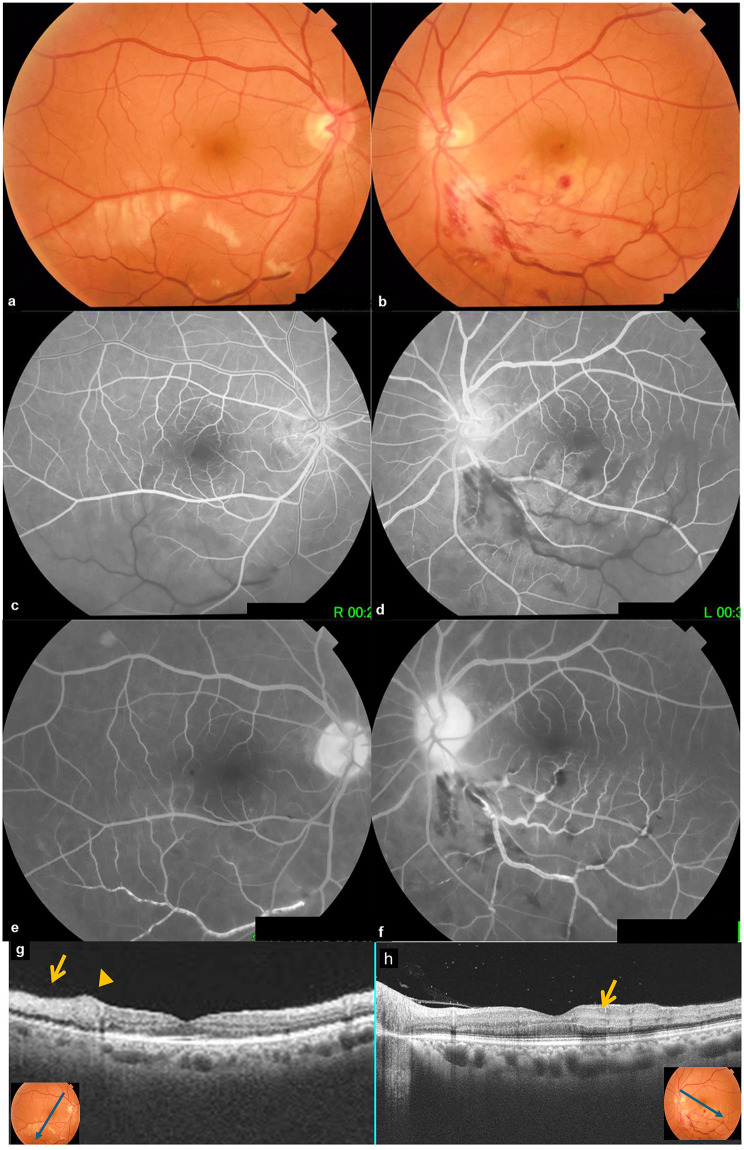
(**a**,** b**) Fundus photograph (**a**, RE; ** b**, LE) showing dilated and tortuous inferotemporal branch retinal vein with associated cotton-wool spots and areas of ischemic retinal whitening in the posterior pole in both eyes and flame-shaped and blot retinal hemorrhages in the LE. **(c**,** d**,** e**,** f)** FA showing a marked delay in retinal arteriovenous transit time and venous filling, with a sluggish flow appearance, and optic disc hyperfluorescence in the late phase, in the affected areas of both eyes. **(g)** OCT scan through whitish retinal lesions in the right eye showing localized thickening and hyperreflectivity of the superficial retinal layers (arrowhead) consisting with cotton-wool spots and a hyperreflectivity of the superficial retinal layers (arrow) secondary to retinal ischemia. **(h)** Macular OCT of the left eye showing a focal retinal thickening and hyperreflective bands in the inner nuclear layer (arrow) consisting with PAMM

Macular OCT of the LE showed focal macular edema and hyperreflective bands at the level of retinal inner nuclear layer consistent with paracentral acute middle maculopathy (PAMM) (Fig. 2). A systemic work-up revealed significantly high blood pressure on blood pressure monitoring. The patient was given antihypertension medication by cardiologist and advised to stop smoking.

**Fig. 3:**
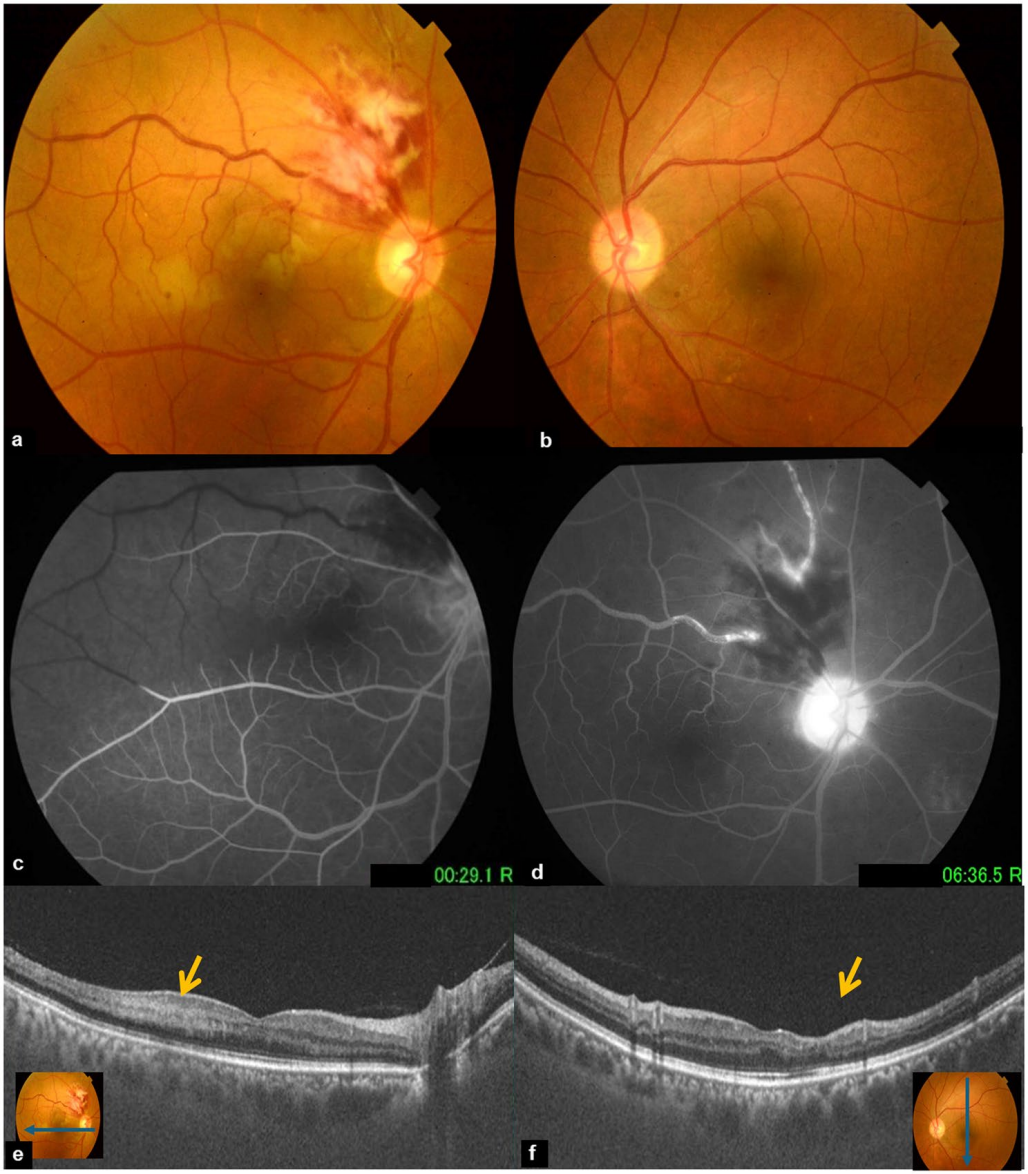
**(a)** Fundus photograph of the RE showing features of a new episode of superotemporal combined BRVO and BRAO including dilated and tortuous veins, retinal hemorrhages, cotton-wool spots, and areas of ischemic retinal whitening in the posterior pole. **(b)** Fundus photograph of the LE showing a complete resolution of features of the previous acute inferotemporal vascular occlusion. **(c**,** d)** Fluorescein angiogram of the right eye showing delayed filling of the superotemporal branch retinal and inferotemporal branch retinal veins, with late vascular leakage and optic disc hyperfluorescence. **(e)** Macular OCT of the right eye showing hyperreflective bands at the level of retinal inner nuclear layer (arrow) consisting with PAMM. **(f)** Macular OCT of the left eye showing temporal macular atrophy (arrow)

Eight months later, the patient was referred again to our department for a painless decrease of vision in the RE. On examination, BCVA was 20/25 in the RE and 20/20 in the LE. Fundus examination showed features of a new episode of superotemporal combined BRVO and BRAO including dilated and tortuous veins, retinal hemorrhages, cotton-wool spots and areas of ischemic retinal whitening in the posterior pole. Features of the previous acute inferotemporal vascular occlusion had completely resolved. Fundus examination of the LE showed a complete resolution of acute findings related to the combined BRVO and BRAO. FA showed delayed filling of the superotemporal branch retinal and inferotemporal branch retinal veins, with late vascular leakage and optic disc hyperfluorescence. Macular OCT of the RE showed PAMM lesions. OCT of the LE showed retinal atrophy of the temporal retina (Fig. 3).

A new, more extensive work-up was performed including a complete blood count, lipid profile, C-reactive protein and erythrocyte sedimentation rate measurements, antinuclear antibodies, anti-neutrophil cytoplasmic antibodies and antiphospholipid antibodies (anticardiolipin antibodies, lupus anticoagulant and β2-glycoprotein-I antibodies), a complete screening for thrombophilic risk factors, including factor V Leiden mutation, protein C, S and antithrombin deficiency, prothrombin gene mutation, homocystinemia, and chest X-Ray. The patient was also reassessed for cardiovascular diseases.

Work-up showed an elevated lupus anticoagulant, which remained positive at a lower titer on repeat testing after three months. Chest X-Ray and chest scan revealed a mass in the left upper pulmonary lobe. The rest of work-up revealed no abnormalities.

Histopathological analysis of the biopsy specimen confirmed the diagnosis of primary lung adenocarcinoma.

**Fig. 4:**
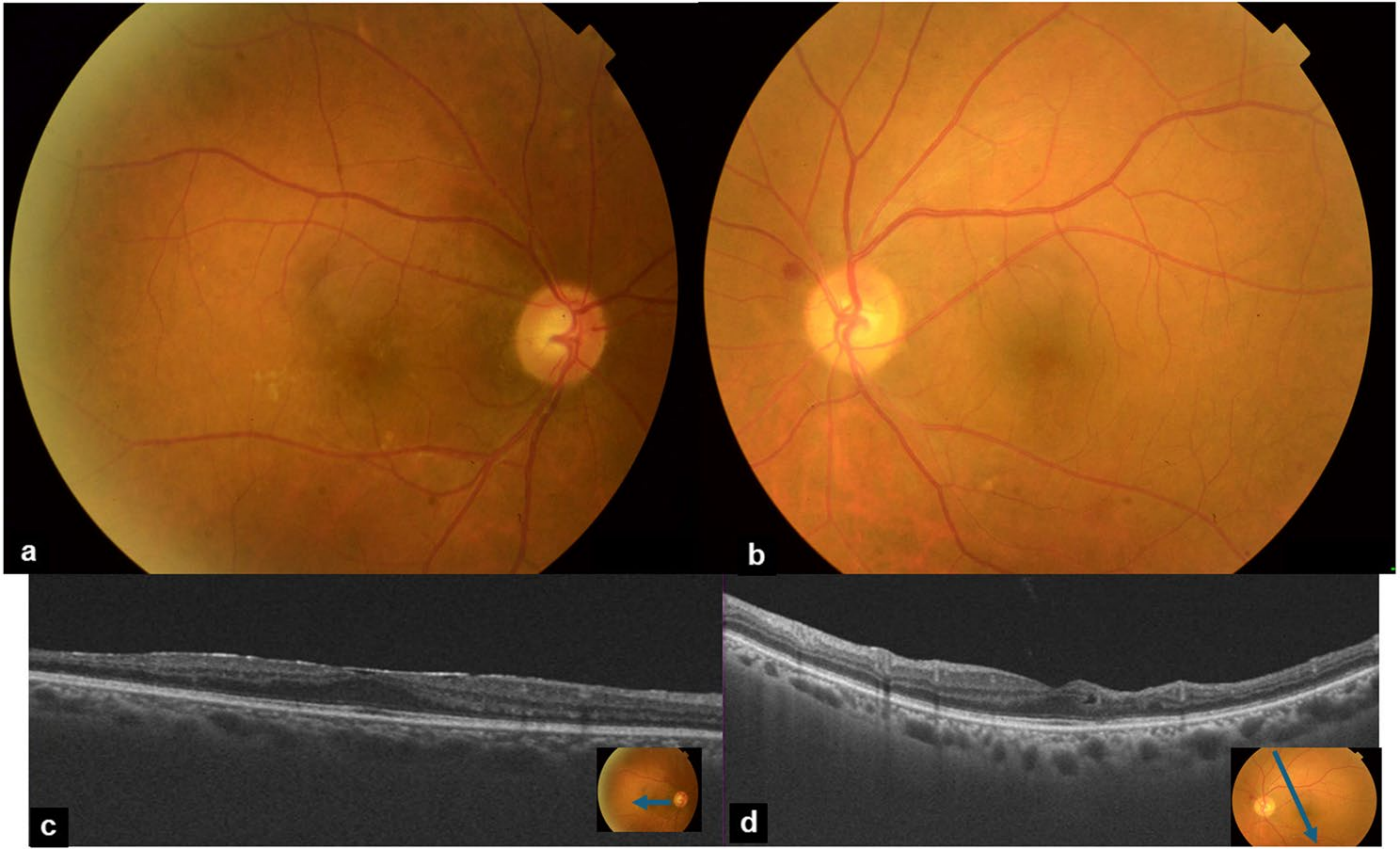
**(a**,** b)** Fundus photograph of the right eye (**a**) and of the left eye (**b**) within normal limits. **(c**,** d)** Swept source OCT demonstrating a diffuse retinal atrophy temporally in both eyes and an epiretinal membrane in the RE (**c**)

The patient subsequently underwent surgical resection of the pulmonary tumor followed by chemotherapy. After a one-year follow-up period, lupus anticoagulant levels had normalized and there was no recurrence of ocular symptoms. At the last ocular examination, BCVA was 20/20 in both eyes. Results of fundus examination were unremarkable. Swept source OCT demonstrated a diffuse retinal atrophy temporally in both eyes and an epiretinal membrane in the RE (Fig. 4).

## Discussion

We report an atypical case of bilateral recurrent BRVO and BRAO with features of PAMM in a 54-year-old male patient with a history of systemic hypertension which ultimately led to the diagnosis of lung adenocarcinoma. To our knowledge, only a few cases of retinal vascular occlusions as the first manifestation of an underlying malignancy have been reported in the literature, and mostly involved central retinal vein occlusion [4–7]. Combined central retinal vein and artery occlusion has been rarely described in association with malignancies [8, 9].

The combination of BRVO and BRAO is an extremely rare event that has been associated with diabetes mellitus, elevated lipids, hypertension, and hyperhomocysteinemia [2, 10].

Retinal vascular occlusion in our patient initially has been linked to age and systemic hypertension. However, a repeat work up showed elevated level of antiphospholipid antibodies, namely lupus anticoaglulant, and a diagnosis of lung adenocarcinoma was ultimately made. After surgical resection of the pulmonary tumor followed by chemotherapy, antiphospholipid antibodies had normalized, and there was no recurrence of vision loss or occlusive retinal events over a one-year follow-up period.

The occurrence of bilateral recurrent combined BRVO and BRAO in our patient probably resulted from a hypercoagulable state due to APS secondary to lung adenocarcinoma.

APS is an autoimmune prothrombotic condition that may occur independently (primary APS) or in association with other systemic diseases (secondary APS). Its pathophysiology involves the binding of antiphospholipid antibodies to cellular antigens, resulting in endothelial activation and thrombus formation [11].

APS has been reported in patients with various solid tumors, with lung carcinoma among the most frequently associated cancers. Studies indicate that patients with lung cancer have a significantly increased risk of developing antiphospholipid antibodies and APS-related thrombotic events compared to the general population [12].

Several mechanisms have been suggested for the association between antiphospholipid antibodies and cancer including the production of autoantibodies by the immune system as a response against tumor antigens, the production of monoclonal immunoglobulins with lupus anticoagulant and anticardiolipin antibody activities and the secretion of anticardiolipin antibodies from tumor cells [12].

BRVO and BRAO have been described in 1% to 2% of patients with APS [13]. A prospective cohort study found that approximately 10% of patients with retinal vein occlusion fulfilled the diagnostic criteria for APS and exhibited a higher prevalence of high-risk antiphospholipid antibody profiles such as isolated lupus anticoagulant positivity or triple positivity (lupus anticoagulant, anticardiolipin antibodies, and anti-β2 glycoprotein I) compared to population-based controls. These findings support the hypothesis that retinal vascular occlusions may, in some cases, represent an organ-specific manifestation of APS [14].

In conclusion, although rare, the association between retinal vascular occlusions and systemic malignancies should not be overlooked. Malignant tumors may induce a prothrombotic state, thereby increasing the risk of thromboembolic events, including BRVO and BRAO. While the precise mechanism linking APS to cancer remains to be clarified, clinicians should consider the possibility of an underlying malignancy in cases of bilateral, recurrent, or combined retinal vascular occlusions, especially when classical cardiovascular risk factors are absent. A thorough and multidisciplinary etiological work-up is essential to identify and manage early potentially life-threatening systemic conditions and related sight-threatening retinal occlusive events.

## Data Availability

Not applicable.
